# Mechanical signatures of left ventricular ejection fraction and mass index in a community cohort (ACE 1950)

**DOI:** 10.1007/s10554-026-03697-7

**Published:** 2026-04-27

**Authors:** Joanna Sulkowska, Julia Brox Skranes, Trygve Berge, Arnljot Tveit, Helge Røsjø, Magnus Nakrem Lyngbakken, Torbjørn Omland, Ulysse Côté-Allard, Siri Lagethon Heck

**Affiliations:** 1 K.G. Jebsen Center for Cardiac Biomarkers, Institute of Clinical Medicine, University of Oslo, Campus Akershus University Hospital, P.b. 1000, NO-1478 Lørenskog, Norway; 2 Department of Diagnostic Imaging, Akershus University Hospital, Lørenskog, Norway; 3 Department of Technology Systems, University of Oslo, Kjeller, Norway; 4 Department of Medical Research, Vestre Viken Bærum Hospital, Bærum, Norway; 5 Department of Cardiology, Oslo University Hospital, Ullevål, Oslo, Norway; 6 Institute of Clinical Medicine, University of Oslo, Oslo, Norway; 7 Akershus Clinical Research Center (ACR), Division of Research and Innovation, Akershus University Hospital, Lørenskog, Norway; 8 Department of Cardiology, Akershus University Hospital, Lørenskog, Norway

**Keywords:** Cardiac magnetic resonance (CMR), Feature tracking (CMR-FT), Strain imaging, Myocardial mechanics, Left ventricular ejection fraction (LVEF), Left ventricular mass index (LVMI), Explainable AI (XAI), Mechanical phenotyping

## Abstract

This study investigated how cardiovascular magnetic resonance feature tracking (CMR-FT)-derived myocardial mechanics, beyond global longitudinal strain (GLS), relate to left ventricular ejection fraction (LVEF) and left ventricular mass index (LVMI). In 186 community-dwelling individuals, global circumferential and longitudinal CMR-FT metrics were extracted, including strain, strain rates, displacement, velocities, time-to-peak measures, mechanical dispersion. Univariate associations with LVEF and LVMI were assessed using Spearman correlation. Multivariable associations between a broad set of mechanical metrics and LVEF and LVMI were evaluated using supervised machine-learning models interpreted with Shapley Additive Explanations (SHAP). Among 17 mechanical metrics, the strongest correlated with LVEF were global circumferential strain (GCS, ρ = -0.71), longitudinal systolic strain rate (sSR_long_, ρ = -0.53), circumferential diastolic strain rate (dSR_circ_, ρ = 0.50), and GLS (ρ = -0.48) (all p < 0.001). In multivariable models, GCS and sSR_long_ remained most influential for LVEF, with GLS dropping to least influential (SHAP rank #17/17). For LVMI, longitudinal diastolic strain rate (dSR_long_) showed the strongest univariate association (ρ = -0.50), followed by GLS (ρ = 0.41) and dSR_circ_ (ρ = -0.40) (all p < 0.001). SHAP ranked dSR_long_ and dSR_circ_ highest, followed by GLS. These findings indicate that feature importance in multivariable SHAP analyses differed from univariate correlation rankings, suggesting that metrics substantially correlated with LVEF or LVMI may contribute less information once myocardial mechanics are considered jointly. LVEF was most strongly associated with GCS and peak systolic strain rate, while LVMI with peak diastolic strain rates and GLS. Future work should test whether integrated mechanical profiles offer added prognostic and diagnostic value beyond single-metric biomarkers.

**Graphical Abstract:**

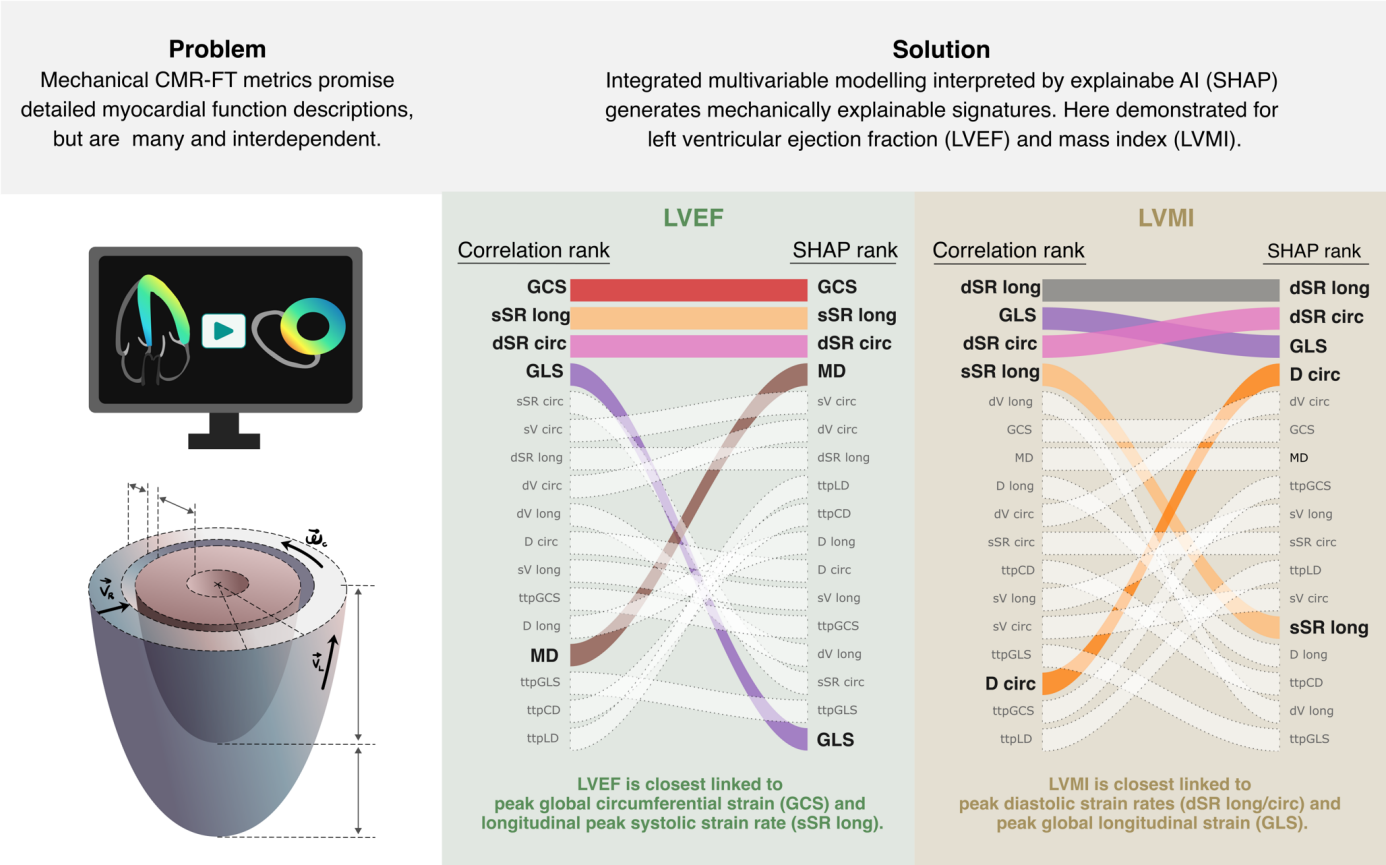

**Supplementary Information:**

The online version contains supplementary material available at 10.1007/s10554-026-03697-7.

## Introduction

Cardiac imaging is routinely used to evaluate *cardiac function*, that is the heart’s ability to adjust its pumping output to meet the body’s changing metabolic needs and maintain homeostasis [1, 2]. In current practice, cardiac function is typically operationalised using left ventricular ejection fraction (LVEF), which describes the proportion of blood ejected from the left ventricle (LV) with each contraction [3]. Volume displacement, however, is a downstream effect of myocardial motion and deformation. Explicit characterisation of myocardial mechanics is therefore hypothesised to improve functional assessment by interrogating processes underlying LVEF. It remains unclear which myocardial mechanics are most informative when these measures are considered jointly rather than individually.

Imaging also captures structural remodelling of the LV, commonly quantified as LV myocardial mass indexed to body surface area (LVMI) [4]. LVMI increases in various contexts and frequently reflects prolonged afterload elevation [5]. As remodelling alters LV geometry, it is plausibly accompanied by systematic changes in myocardial mechanics. Characterising how myocardial mechanics relate to LVMI may provide insight about mechanical characteristics accompanying structural remodelling.

Cardiac magnetic resonance (CMR) allows reference-standard quantification of LVEF and LVMI, as well as quantification of myocardial motion and deformation through CMR feature tracking (CMR-FT). In practice, the myocardial mechanical characteristics are often distilled to a single headline metric: peak global longitudinal strain (GLS), which has been shown to provide diagnostic and prognostic insights when derived from both echocardiography [6] and CMR [7–10]. While GLS captures longitudinal deformation, it cannot be assumed to reflect deformation in other spatial directions and omits rate and timing properties that may provide complementary information about both contraction and relaxation. CMR-FT provides multiple additional mechanical metrics beyond GLS, including peak circumferential and radial strain (GCS, GRS), displacement (D), systolic/diastolic strain rates (sSR, dSR) and velocities (sV, dV), time-to-peak measures of global strain/displacement (ttpGS, ttpD), and mechanical dispersion (MD). How a multidirectional mechanical panel relates to LVEF and LVMI remains incompletely characterised, particularly when considered jointly. Although LVEF is an imperfect summary of systolic function, it remains the most widely used clinical descriptor and, together with LVMI, serves as a pragmatic anchor for analysing how broader myocardial mechanics relate to function and remodelling. Because LVEF reflects the integrated consequence of myocardial deformation, and LVMI reflects global remodelling, examining mechanics jointly may be informative beyond single-metric associations, contextualising them as a set. Studying these relationships in community-dwelling older adults characterises these associations in the normal-to-early disease spectrum, providing a reference for later disease and prognostic studies.

Accordingly, this study examines how a broad panel of global CMR-FT mechanical metrics is associated with two established CMR-derived measures of LV function and structure, LVEF and LVMI, in community-dwelling older adults. The aims are (i) to quantify the association of global CMR-FT mechanical metrics with LVEF and LVMI, and (ii) to identify which metrics contribute most consistently when the mechanical profile is considered jointly.

## Method

### Ethical approval

The study adhered to the Declaration of Helsinki and was approved by the Regional Committee for Medical and Health Research Ethics in Norway (ref.2011/1475). All participants provided informed consent.

### Study population

**Fig. 1:**
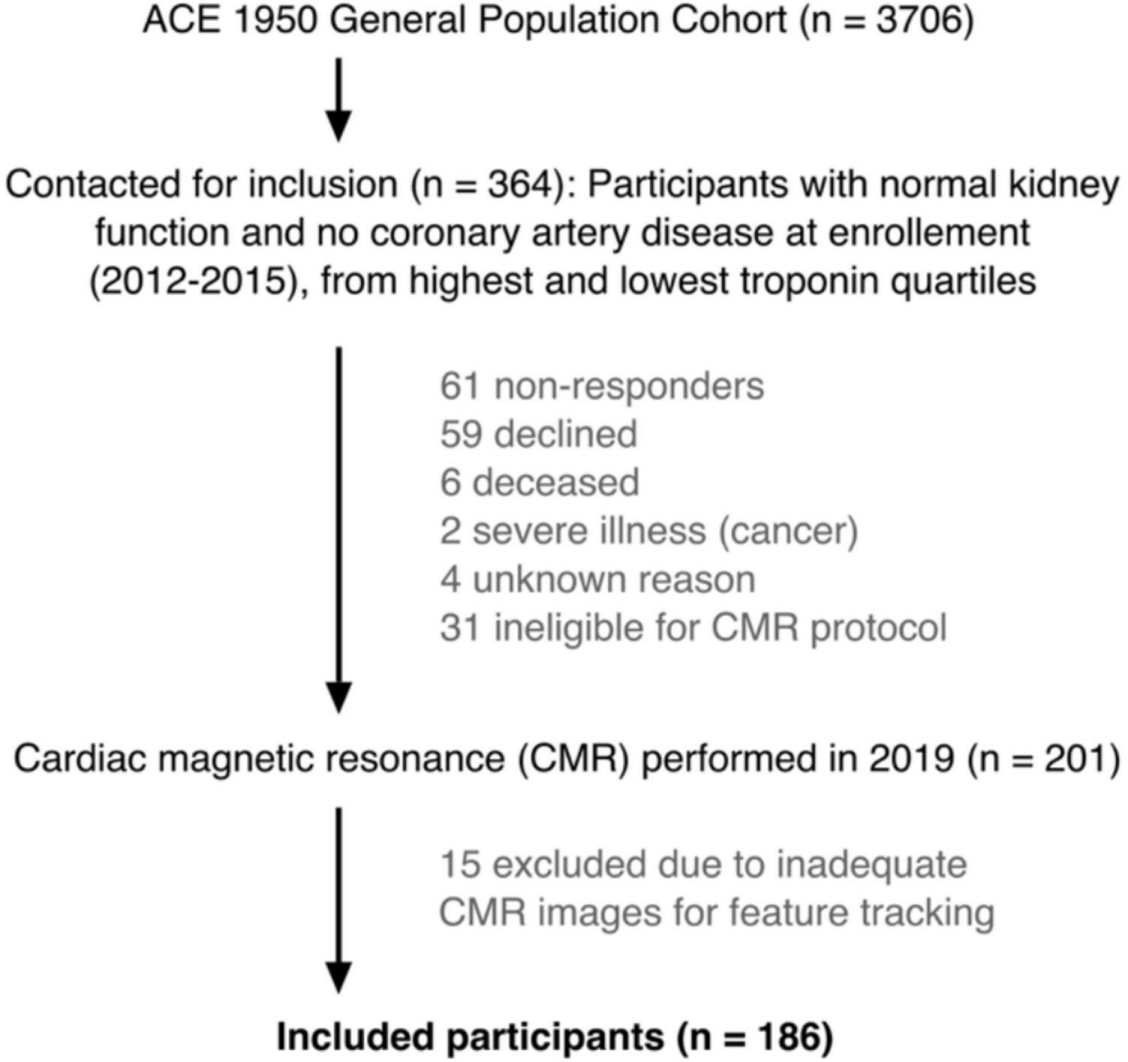
Study population

The study population comes from the Akershus Cardiac Examination 1950 (ACE 1950) Study, comprising individuals from the general population born in 1950 residing in Akershus County (Norway), which investigates the development and progression of cardiovascular disease [11]. A subgroup of participants (n = 363) was invited to undergo CMR if they, at enrolment between 2012 and 2015, had normal kidney function, no history of coronary artery disease, and cardiac troponin I levels in the highest or lowest cohort quartiles (Fig. 1). CMR was performed in 2019 (n = 201). Participants with inadequate images for CMR-FT or for quantification of LVEF and LVMI were excluded from the analyses (n = 186 included in analyses).

### CMR acquisition and specifications

CMR images were acquired using a 1.5T Philips Achieva MRI scanner. Short-axis images covering the ventricles and single-slice long-axis views in the four-chamber, two-chamber, and LV outflow tract orientations were acquired with balanced breath-hold steady-state free precession (bSSFP) cine sequences. Each sequence captured 30 frames per cardiac cycle with a slice thickness of 8 mm, flip angle of 45 degrees, echo time of 1.53 ms, repetition time of 3.1 ms, and field of view of 30 × 30 cm with a matrix of 240 × 240 voxels (1.25 × 1.25 x 8 mm^3^ per voxel).

### Extraction of mechanical metrics by CMR-FT

**Fig. 2:**
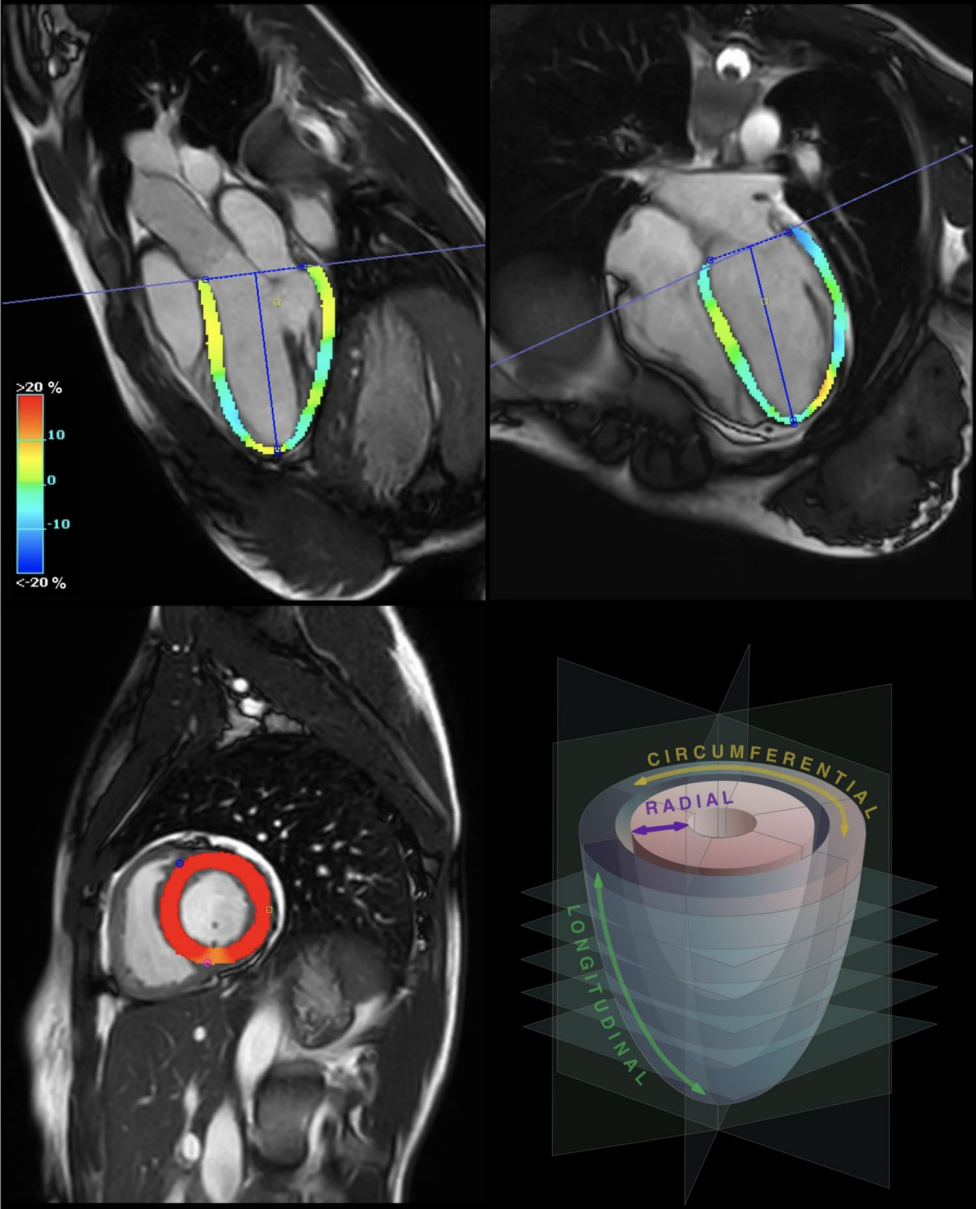
Extraction of myocardial mechanical metrics with CMR-FT. Colour overlays illustrate deformation in the longitudinal (top panels) and radial (bottom left) directions relative to end-diastole. Bottom right shows a schematic of the LV myocardium with denoted spatial directions

Metrics collectively describing global LV myocardial mechanics, were extracted from the above described bSSFP images using CMR-FT of the clinical post-processing software CVI42 (v5.13.5, Circle Cardiovascular Imaging Inc.). Spatial directions are illustrated in Fig. 2, and metric definitions provided in Table 1. The following metrics were extracted in the longitudinal, circumferential and radial spatial directions: peak strain (GLS, GCS, GRS), peak displacement (D), peak systolic/diastolic strain rates (sSR, dSR) and velocities (sV, dV), time-to-peak strain and displacement in all spatial directions (ttpGLS, ttpGCS, ttpGRS, ttpLD, ttpCD, ttpRD) along with mechanical dispersion (MD). MD was calculated as standard deviation of segmental time-to-peak longitudinal strain across 16 AHA myocardial segments [12].

**Table 1:** Descriptions of the 25 global left ventricular myocardial mechanical metrics derived by CMR-FT

Metric	Description, unit	Directions	Abbreviation	Higher reported absolute values indicate	Included in multivariable model
Peak global strain	Maximal deformation relative to end-diastole, %	Longitudinal	GLS	Greater shortening ^a^	Yes
		Circumferential	GCS	Greater shortening ^a^	Yes
		Radial	GRS	Greater thickening	Sensitivity analysis
Peak global systolic strain rate	Maximal deformation rate in systole, 1/s	Longitudinal	sSR_long_	Greater deformation rate during contraction ^a^	Yes
		Circumferential	sSR_circ_	Greater deformation rate during contraction ^a^	Yes
		Radial	sSR_rad_	Greater deformation rate during contraction	Sensitivity analysis
Peak global diastolic strain rate	Maximal deformation rate in diastole, 1/s	Longitudinal	dSR_long_	Greater deformation rate during relaxation	Yes
		Circumferential	dSR_circ_	Greater deformation rate during relaxation	Yes
		Radial	dSR_rad_	Greater deformation rate during relaxation ^a^	Sensitivity analysis
Peak global displacement	Maximal global displacement relative to end-diastole, mm (longitudinal and radial), deg (circumferential)	Longitudinal	D_long_	Greater displacement	Yes
		Circumferential	D_circ_	Greater displacement	Yes
		Radial	D_rad_	Greater displacement	Sensitivity analysis
Peak global systolic velocity	Maximal global displacement rate (velocity) in systole, mm/s (longitudinal and radial), deg/s (circumferential)	Longitudinal	sV_long_	Greater displacement rate during contraction	Yes
		Circumferential	sV_circ_	Greater displacement rate during contraction	Yes
		Radial	sV_rad_	Greater displacement rate during contraction	Sensitivity analysis
Peak global diastolic velocity	Maximal global displacement rate (velocity) in diastole, mm/s (longitudinal and radial), deg/s (circumferential)	Longitudinal	dV_long_	Greater displacement rate during relaxation ^a^	Yes
		Circumferential	dV_circ_	Greater displacement rate during relaxation ^a^	Yes
		Radial	dV_rad_	Greater displacement rate during relaxation ^a^	Sensitivity analysis
Time to peak global strain	Time to maximal global deformation, ms	Longitudinal	ttpGLS	Longer time to maximal deformation	Yes
		Circumferential	ttpGCS	Longer time to maximal deformation	Yes
		Radial	ttpGRS	Longer time to maximal deformation	Sensitivity analysis
Time to peak global displacement	Time to maximal global displacement, ms	Longitudinal	ttpLD	Longer time to maximal displacement	Yes
		Circumferential	ttpCD	Longer time to maximal displacement	Yes
		Radial	ttpRD	Longer time to maximal displacement	Sensitivity analysis
Mechanical dispersion	Standard deviation of segmental time to peak longitudinal strain, ms	Longitudinal	MD	Greater temporal heterogeneity between segmental shortening	Yes

Time is referenced to end-diastole. CMR-FT: cardiac magnetic resonance feature tracking.

^a^ Metrics that are negative by convention are reported as magnitudes/absolute values to aid readability (even though model training was done on signed values): GCS, GLS, sSR_circ_, sSR_long_, dSR_rad_, dV_rad_, dV_circ_, dV_long._

End-diastolic myocardial contours were semi-automatically placed slightly within the myocardium to reduce tracking errors [13]. Tracking throughout the cardiac cycle was visually quality-checked by a radiology resident. Radial and circumferential metrics were derived from the short-axis stack and longitudinal from the three long-axis views. Included participants had complete bSSFP series with adequate coverage of all 16 AHA myocardial segments, and no major tracking errors.

### Sign conventions of mechanical metrics

Correlation analyses and model training were performed on signed data. When reporting medians and IQRs for metrics negative by convention (GCS, GLS, sSR_circ_, sSR_long_, dSR_rad_, dV_rad_, dV_circ_, dV_long_), we show |median| with |IQR bounds| were low and high bounds are reordered (not median (|x|) or IQR (|x|)). For readability, descriptive results (“greater”/”lower”) are presented as magnitudes/absolute values, correlation coefficients are reported with the native sign.

### Reference LVMI and LVEF

**Fig. 3:**
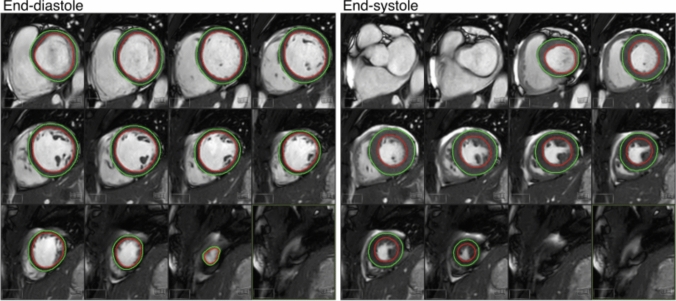
Reference LVEF and LVMI were derived from CMR short-axis cine images

Reference LVMI (g/m^2^) and LVEF (%) were quantified semi-automatically from short-axis SSFP CMR images by a cardiothoracic radiologist using the CVI42 post-processing software (Fig. 3). Epicardial and endocardial contours were placed at end-systole and end-diastole, trabeculations and papillary muscles were included in cavity volumes and excluded from myocardial mass. LVMI was derived from end-diastolic myocardial volume (epicardial-endocardial difference) multiplied by myocardial density and indexed to body surface area. LVEF was calculated as percent reduction in LV cavity volume from end-diastole to end-systole, based on endocardial contours.

### Univariate associations

Spearman rank correlation (ρ) quantified univariate associations between global CMR-FT metrics and cine-derived LVEF and LVMI. Correlation coefficients with p-values were reported and ranked after absolute value of ρ for each endpoint.

### Inter-directional dependence and feature selection for multivariable models

**Fig. 4:**
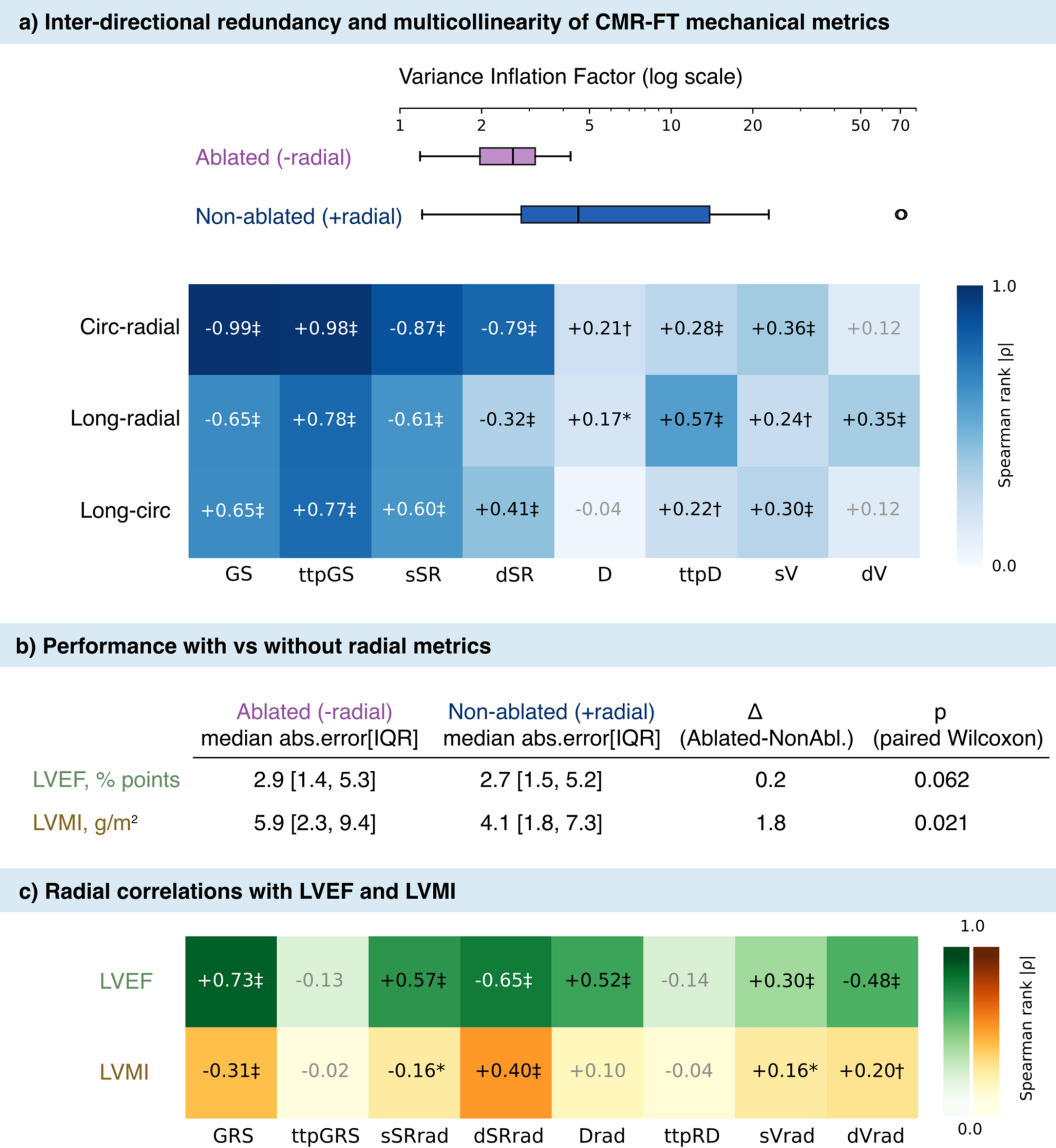
Radial-metric redundancy and sensitivity analysis. (**a**) Variance inflation analysis for mechanical metrics in primary models excluding (-radial) and including (+ radial) metrics. Inter-directional Spearman correlations between circumferential-radial, longitudinal-radial, and longitudinal-circumferential variants of mechanical classes. (**b**) Out-of-training-sample median absolute error (IQR) across 50 repeats for primary (-radial) and sensitivity (+ radial) models estimating LVEF and LVMI, p-values from paired Wilcoxon signed-rank tests. (**c**) Spearman correlations between radial metrics and LVEF and LVMI. In panels a and c shade encodes |ρ|, annotations show signed ρ (*p < 0.05, †p < 0.01, ‡p < 0.001). GS, peak global strain. ttpGS, time-to-peak global strain. sSR/dSR, peak systolic/diastolic strain rate. D, displacement. ttpD, time-to-peak displacement. sV/dV, peak systolic/diastolic velocity. R/rad denotes radial variants (GRS/ttpGRS/sSRrad/dSRrad/Drad/ttpRD/sVrad/dVrad)

Pairwise Spearman rank correlations between circumferential, longitudinal, and radial metrics within the same mechanical descriptor class (peak strain, displacement, systolic/diastolic strain rates and velocities, and time-to-peak measures) characterised redundancy across spatial directions. Variance inflation factors (VIF) assessed multicollinearity among CMR-FT metrics. Correlation analysis indicated substantial inter-directional dependence, with GCS and GRS showing near-perfect correlation (ρ = 0.99, p < 0.001). Given elevated VIFs when radial, circumferential, and longitudinal metrics were considered jointly, and limited reported reproducibility of radial strain by prior studies [14, 15], radial metrics were excluded from primary multivariable models to prioritise interpretability. Redundancy patterns and sensitivity analyses including radial metrics are described in the Results (Fig. 4).

### Multivariable modelling of LVMI and LVEF from global myocardial mechanics

LVMI and LVEF were modelled as separate supervised regression tasks from global CMR-FT myocardial mechanical metrics (Table 1). Primary models used a total of 17 longitudinal and circumferential CMR-FT metrics. Sensitivity models including radial metrics (25 metrics in total) evaluated the interpretability-precision trade-off (Fig. 4). The input metrics were standardised before modelling and followed sign conventions.

### Model training and evaluation

Given the sample size, we used nested cross-validation to separate hyperparameter tuning from model assessment and to estimate out-of-sample performance. Of 186 participants, 10 were set aside for exploratory analyses and were never evaluated. The remaining 176 participants were assigned to five outer folds; in each iteration, 35 participants formed the test set and the remaining participants, together with the 10 exploratory cases (n = 151), formed the training pool. Hyperparameters were selected within the training pool using three-fold inner cross-validation across six regression models (random forest, support vector machine, neural network, linear regression, ridge, and lasso), tuned via randomised search over 100 configurations [16]. For each outer fold, the best inner-loop configuration was refit on all 151 training cases and evaluated on the held-out test fold. Aggregating across outer folds yielded out-of-sample evaluations for 175 unique participants with strict train-test separation.

To reduce stochastic variability (e.g., random initialisation), we repeated the full training procedure 50 times with distinct random seeds. We report averages across runs (including the sign of the association) to improve stability and reduce sensitivity to random fluctuations. All models were trained from scratch without pretraining.

### Model performance

Agreement between reference LVMI and LVEF and model estimates on pooled outer-fold predictions (n = 175) was evaluated with Bland–Altman analysis with reported bias and limits of agreement (bias ± 1.96 SD) approximating the range within which ∼95% of differences are expected to lie. Median absolute error and root mean squared error (RMSE) was calculated and benchmarked against published CMR expert agreement [17].

### Permutation test

To assess whether models were learning genuine feature-outcome relationships rather than random patterns, permutation tests were performed. LVMI and LVEF were randomly shuffled across participants and models retrained using the same nested cross-validation approach.

### Explainable AI derived mechanical phenotypes of LVMI and LVEF

After training, machine learning models were interpreted using the explainable AI (XAI) method SHapley Additive exPlanations (SHAP) [18]. For each participant and endpoint (LVMI, LVEF), the participant-level SHAP value for each mechanical metric quantifies how that metric’s standardised input value shifts the model estimate relative to the baseline (expected) value: a positive participant-level SHAP value for a metric indicates that it increases the estimate, while negative SHAP indicates it decreases the estimate for that person.

For each of the 50 independent training/evaluation runs, the importance of each CMR-FT metric was summarised by computing the mean absolute SHAP value across participants for that metric, yielding 50 ranks for each metric. The mean rank across 50 runs determined the overall metric-importance for estimating LVEF and LVMI when considered jointly with the other mechanical metrics. This SHAP-ranked constellation of metrics most consistently driving the models’ estimates of LVEF and LVMI were defined as the mechanical phenotypes for each endpoint.

### Complementarity to clinical variables

Potential incremental information of CMR-FT beyond clinical variables and cardiovascular risk factors was assessed by unsupervised feature exploration and supervised incremental-value models.

First, topological data analysis (Mapper, previously described [19, 20]) visualised overlap between standardised radial, circumferential, longitudinal CMF-FT metrics and clinical variables: age, sex, diabetes mellitus, BMI, BSA, hypertension, smoking status. Features (25 mechanical + 7 clinical) were embedded to 3D with t-SNE, partitioned into 8 overlapping bins (2 × 2 × 2 with 50% overlap), clustered within bins using k-means (k = 5), then connected into a network where nodes represent feature clusters and edges indicate shared features between clusters. Nodes were coloured by the proportion of clinical variables in the cluster, allowing assessment of complementarity versus overlap between mechanical and clinical information.

Secondly, additional models were trained using clinical variables alone and clinical variables combined with CMR-FT metrics to quantify incremental information of CMR-FT (Supplementary methods).

### Software for data analysis and machine learning

Analyses were conducted in Python v3.8.5. Scikit-learn v1.0 was used for supervised machine learning, SciPy v1.10.1 for statistical analyses, pandas v1.2.0 and NumPy v1.20.0 for data handling, matplotlib v3.3.4 and seaborn v0.11.2 for visualisation, SHapleyAdditiveExPlanations (SHAP) v0.44.1 was used for model interpretation, unsupervised feature exploration used KeplerMapper v2.0.1. Inkscape v1.3.2 was used to for illustrations and harmonise layout/typography of Python-generated plots, edits were cosmetic only.

### Quality assessment

The study adhered to key recommendations of the Proposed Requirements for Cardiovascular Imaging-Related Machine Learning Evaluation (PRIME) checklist [21]. The manuscript benefited from ChatGPT (OpenAI) for grammar and clarity.

## Results

### Study population characteristics

Individual CMR data from 186 participants were analysed (Fig. 1). Clinical, CMR characteristics, and CMR-FT metrics are summarised in Tables 2, 3, 4.

**Table 2:** Clinical characteristics

Number of participants	186
Female, number (%)	89 (47.8%)
Age, years	69.0 [68.6, 69.2]
Hypertension, number (%)	112 (60.2%)
Body mass index, kg/ m^2^	26.2 [23.5, 28.5]
Body surface area, m^2^	1.9 [1.8, 2.1]
Diabetes mellitus, number (%)	9 (4.8%)
Daily smokers, number (%)	10 (5.4%)
Caucasians, number (%)	185 (99%)
Higher education, number (%)	99 (53%) ^a^
Inclusion site Akershus University Hospital (vs Bærum Hospital), number (%)	122 (66%)
eGFR mL/min/1.73 m2	92.6 [85.2, 95.5] ^a^
Coronary artery disease, number (%)	10 (5.4%)
Heart failure, number (%)	6 (3.2%) ^b^
Systolic blood pressure, mmHg	136 [124, 148]
Diastolic blood pressure, mmHg	75 [70, 82]
Heart rate, bpm	65 [58, 71]
HbA1c, mmol/mol	37.2 [35.0, 40.0] ^a^
Total cholesterol, mmol/L	5.4 [4.8, 6.0] ^a^
High-density lipoprotein, mmol/L	1.6 [1.3, 1.9] ^a^
Triglycerides, mmol/L	1.2 [0.9, 1.6] ^a^
Cardiac troponin T, ng/L	7.0 [5.0, 12.6]

Categorical variables in numbers and percent of all study participants, continuous as median and interquartile interval. ^a^ one participant with missing data. ^b^ three participants with missing data.

**Table 3:** Left ventricular characteristics by CMR

LV mass (LVM), gram	88.5 [73.9, 111.3]
LV mass indexed by BSA, gram/m^2^	41.8 [34.9, 52.5]
LV ejection fraction (LVEF), %	59.9 [55.1, 64.2]
LV end-diastolic volume indexed by BSA, mL/m^2^	69.7 [58.4, 80.0]
LV end-systolic volume indexed by BSA, mL/m^2^	26.7 [21.5, 34.7]
Median and interquartile interval. BSA: body surface area

**Table 4:** Global deformation metrics of the left ventricle

	Radial	Circumferential	Longitudinal
Peak strain (ɛ), %	28.7 [24.5, 32.9]	17.6 [16.0, 19.2]	17.25 [15.8, 18.7]
Time to peak strain (ttpS), ms	359.4 [330.7, 390.4]	359.9 [332.0, 393.0]	359.2 [335.1, 396.9]
Peak systolic strain rate (sSR), %/s	1.4 [1.2, 1.7]	0.9 [0.8, 1.0]	0.8 [0.7, 0.9]
Peak diastolic strain rate (dSR), %/s	1.3 [1.0, 1.6]	0.7 [0.6, 0.8]	0.8 [0.7, 0.9]
Peak displacement (D), mm (radial, longit.), degrees (circumf.)	5.0 [4.4, 5.4]	1.9 [0.9, 2.8]	4.8 [4.0, 5.8]
Time to peak displacement (ttpD), ms	360.2 [330.5, 395.9]	375.2 [311.5, 448.1]	372.7 [339.0, 400.6]
Peak systolic velocity (sV), mm/s (radial, longit.), deg/s (circumf.)	24.9 [22.1, 28.2]	18.7 [13.0, 26.1]	26.6 [22.5, 31.7]
Peak diastolic velocity (dV), mm/s (radial, longit.), deg/s (circumf.)	21.7 [18.7, 24.3]	14.0 [8.9, 19.0]	28.9 [24.3, 33.7]
Mechanical dispersion (MD), ms	Not applicable	Not applicable	85.9 [69.4, 110.3]

Global mechanical metrics of the left ventricle used in models are described as median and interquartile range (IQR). GCS, GLS, sSR_circ_, sSR_long_, dSR_rad_, dV_rad_, dV_circ_, dV_long_ are by convention negative, but for readability shown as |median| with |IQR bounds| where low and high bounds are reordered (not median (|x|) or IQR (|x|)). Model training and descriptive statistics were performed on the signed values.

### Inter-directional redundancy and sensitivity to radial-feature inclusion

Several mechanical classes showed substantial inter-directional correlation (Fig. 4a). Circumferential-radial correlations were particularly high for GCS and GRS (ρ = -0.99, p < 0.001) and for ttpGCS vs ttpGRS (ρ = 0.98, p < 0.001). Consequently, VIFs were higher when radial, circumferential, and longitudinal metrics were jointly included than among circumferential and longitudinal only. Accordingly, primary multivariable models were trained using only circumferential and longitudinal CMR-FT metrics to prioritise interpretability. Adding radial metrics significantly reduced median absolute error for LVMI by 1.8 g/m^2^ (p = 0.021, by paired Wilcoxon signed rank test) versus the primary (without radial metrics), but non-significantly for LVEF (reduction by 0.2 %-points, p = 0.062), reflecting a modest precision gain for LVMI with lower interpretability (Fig. 4b). Univariate associations between radial metrics and LVEF/LVMI are shown in Fig. 4c.

## Associations between CMR-FT mechanics and LVEF/LVMI: univariate correlations and multivariable feature importance (SHAP)

**Fig. 5:**
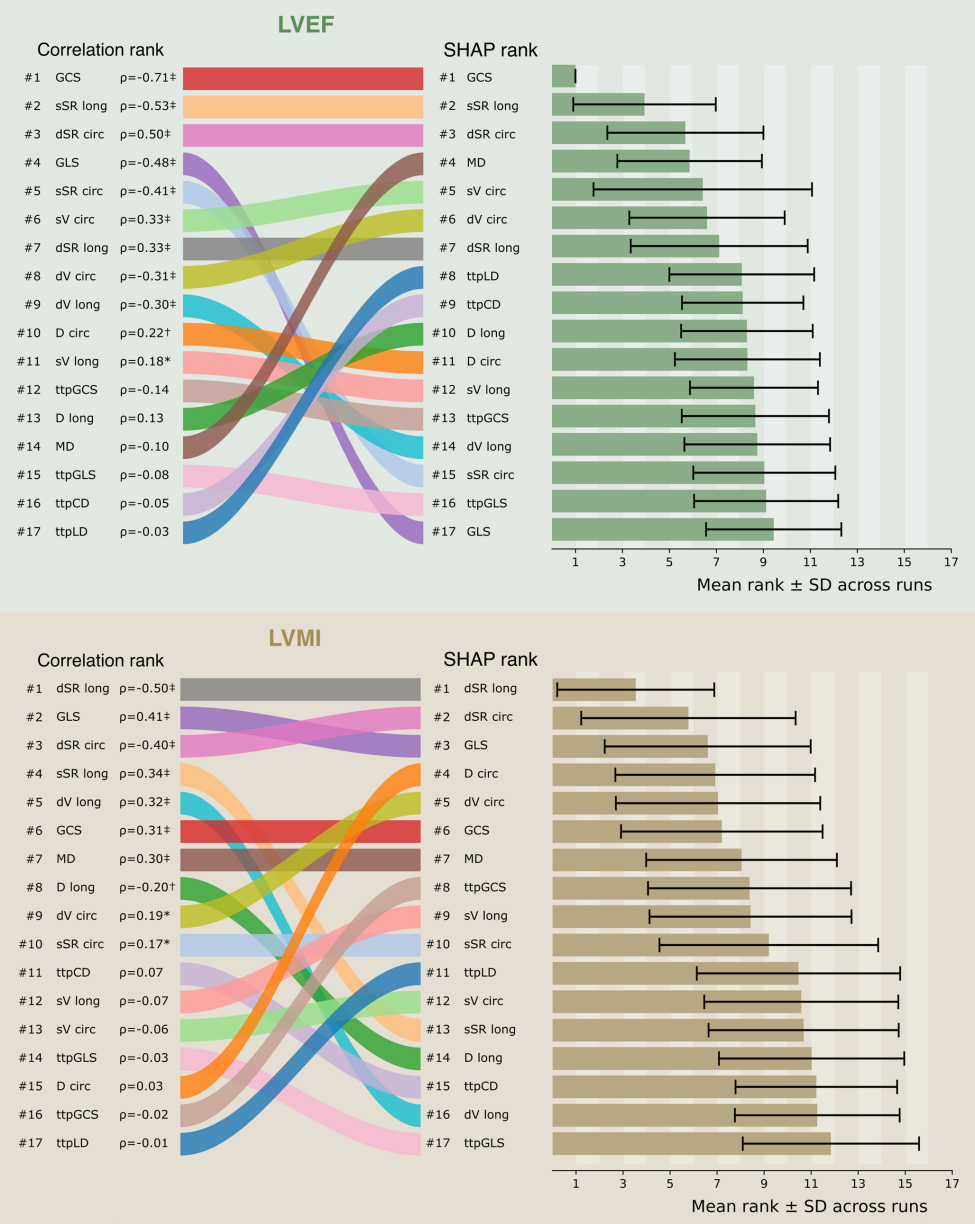
Univariate versus joint importance of global longitudinal and circumferential CMR-FT mechanics for LVEF and LVMI. Rank-flow comparison of univariate and multivariate associations, highlighting rank shifts when mechanics are considered jointly. Univariate importance ranking is done by absolute Spearman correlation (ρ), and re-ranking by mean absolute SHAP value across 50 refit multivariable models. *p < 0.05, †p < 0.01, ‡p < 0.001

Figure 5 displays univariate correlations of longitudinal and circumferential CMR-FT metrics, and their relative rankings from multivariable modelling using SHAP analysis. For LVMI and LVEF, metrics are ranked by absolute Spearman correlation with LVEF/LVMI and then re-ranked by mean absolute SHAP value from multivariable models (across 50 repeated fits), illustrating how importance changes when mechanics are considered jointly.

**Fig. 6:**
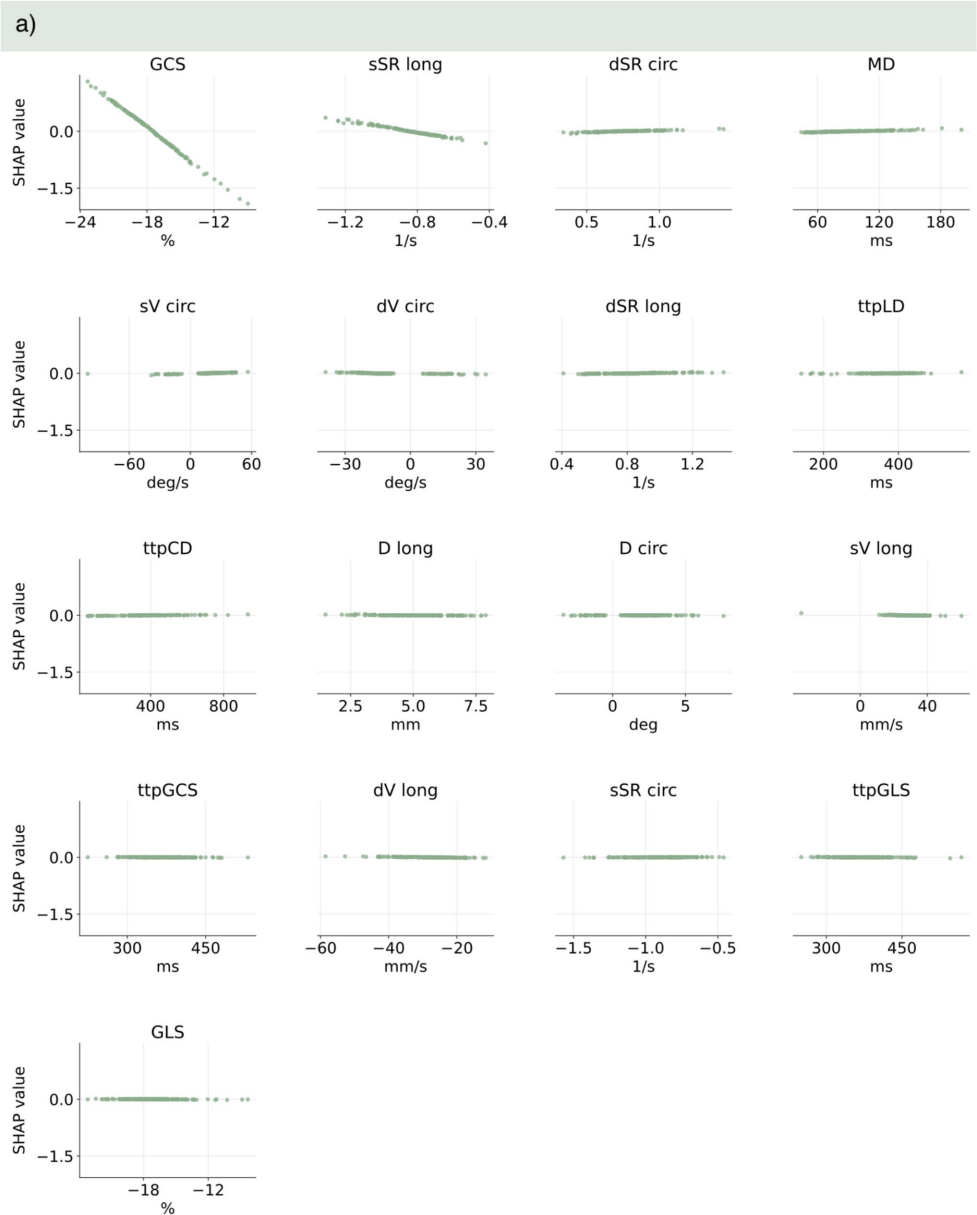

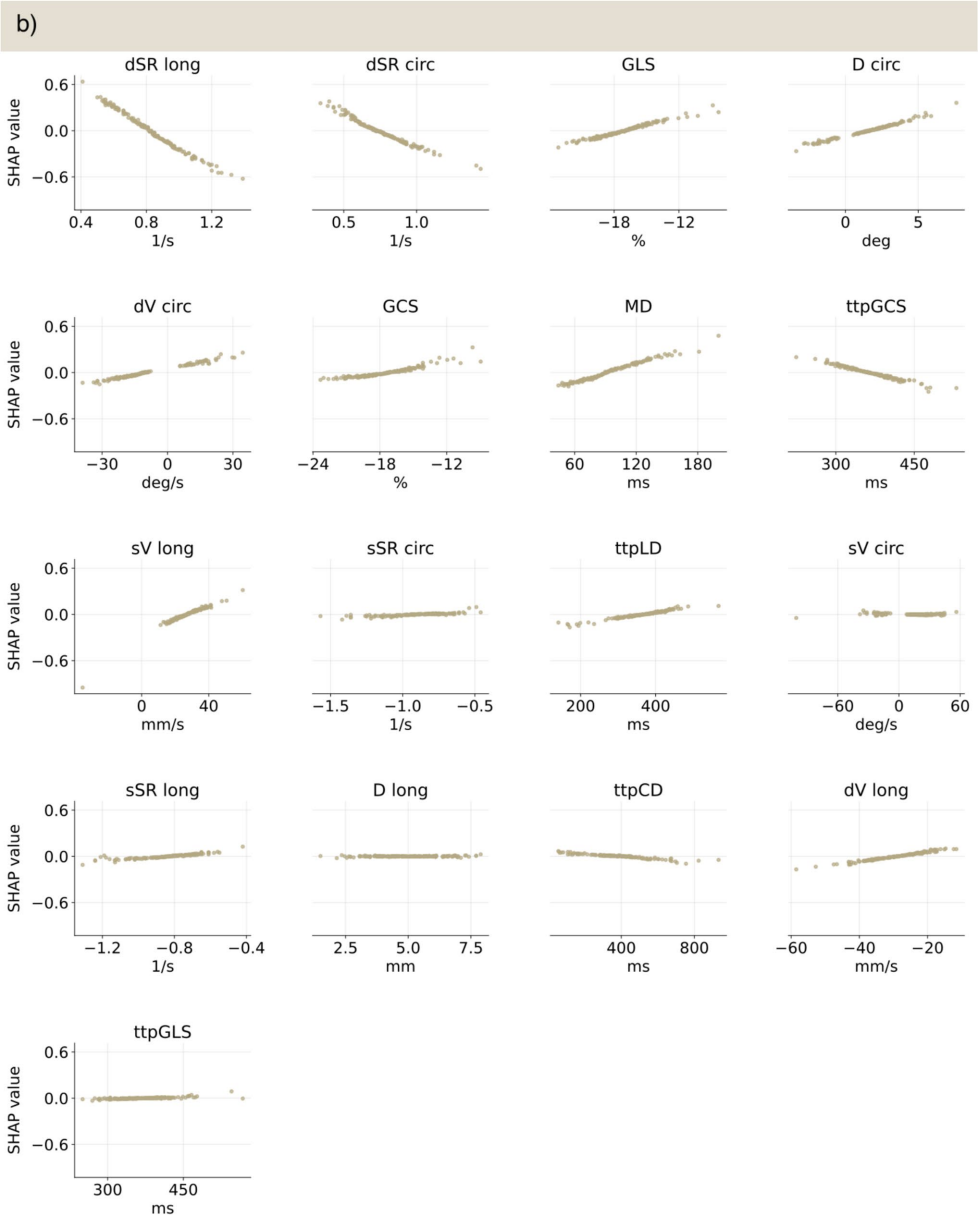
SHAP-based direction and magnitude of association between circumferential and longitudinal CMR-FT metrics and (**a**) LVEF, (**b**) LVMI in machine-learning models. Features are ordered by mean absolute SHAP value (global association with LVEF/LVMI). Points represent participants (n = 175, pooled-outer-test-fold) with the x-axis representing the CMR-FT metric value and the y-axis the associated SHAP value for standardised outcome averaged across 50 fits. Positive SHAP indicates increase in estimated LVEF/LVMI relative to baseline, negative decrease. Clear monotonic slopes indicate consistent association between CMR-FT metric and LVEF/LVMI, near-horizontal cloud around zero indicate limited contribution. Vertical spread of SHAP values reflects metric impact across participants (larger spread = greater influence)

For LVEF, the strongest correlations were observed for GCS (ρ = -0.71, p < 0.001), longitudinal systolic strain rate (sSR_long_, ρ = -0.53, p < 0.001), circumferential diastolic strain rate (dSR_circ_, ρ = 0.50, p < 0.001), and global longitudinal strain (GLS, ρ = -0.48, p < 0.001). In multivariable models, GCS, sSR_long_, and dSR_circ_ remained the highest-ranked features by mean |SHAP|, whereas GLS dropped to the lowest-ranked feature (#17/17). Across 50 fits, average SHAP calculations indicated that higher |GCS| and |sSR_long_| showed the most consistent participant-level associations with higher LVEF (clear monotonic trends with substantial SHAP spread), whereas SHAP values of other metrics clustered closer to zero, indicating limited contribution (Fig. 6a).

For LVMI, longitudinal diastolic strain rate (dSR_long_) had the strongest correlation (ρ = -0.50, p < 0.001), followed by GLS (ρ = 0.41, p < 0.001), dSR_circ_ (ρ = -0.40, p < 0.001), and sSR_long_ (ρ = 0.34, p < 0.001). Upon multivariate modelling dSR_long_, dSR_circ_ and GLS remained among the top 3 ranked metrics. Figure 6b shows association stability across 50 repeated fits: higher LVMI corresponded to lower dSR_long_ and dSR_circ_, and higher |GLS|.

**Fig. 7:**
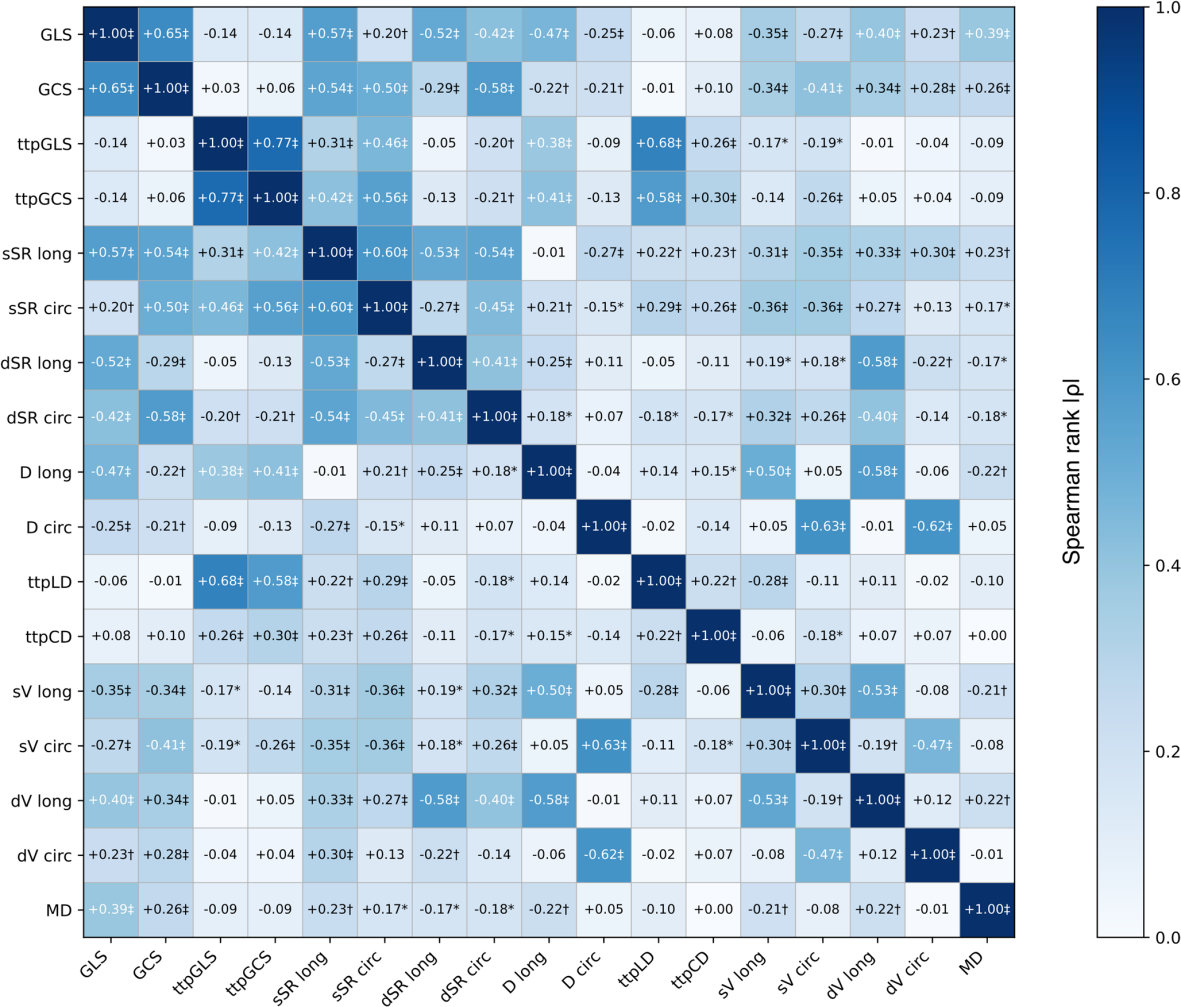
Pairwise correlations between the 17 circumferential and longitudinal CMR-FT metrics of the primary multivariable models. Shade encodes Spearman rank correlation magnitude (|ρ|) annotated with signed correlation coefficient (ρ). *p < 0.05, †p < 0.01, ‡p < 0.001

The correlation structure among the 17 circumferential/longitudinal CMR-FT metrics in the primary models is shown in Fig. 7, contextualising multivariable modelling.

### Multivariate model agreement with cine-derived reference values

**Fig. 8:**
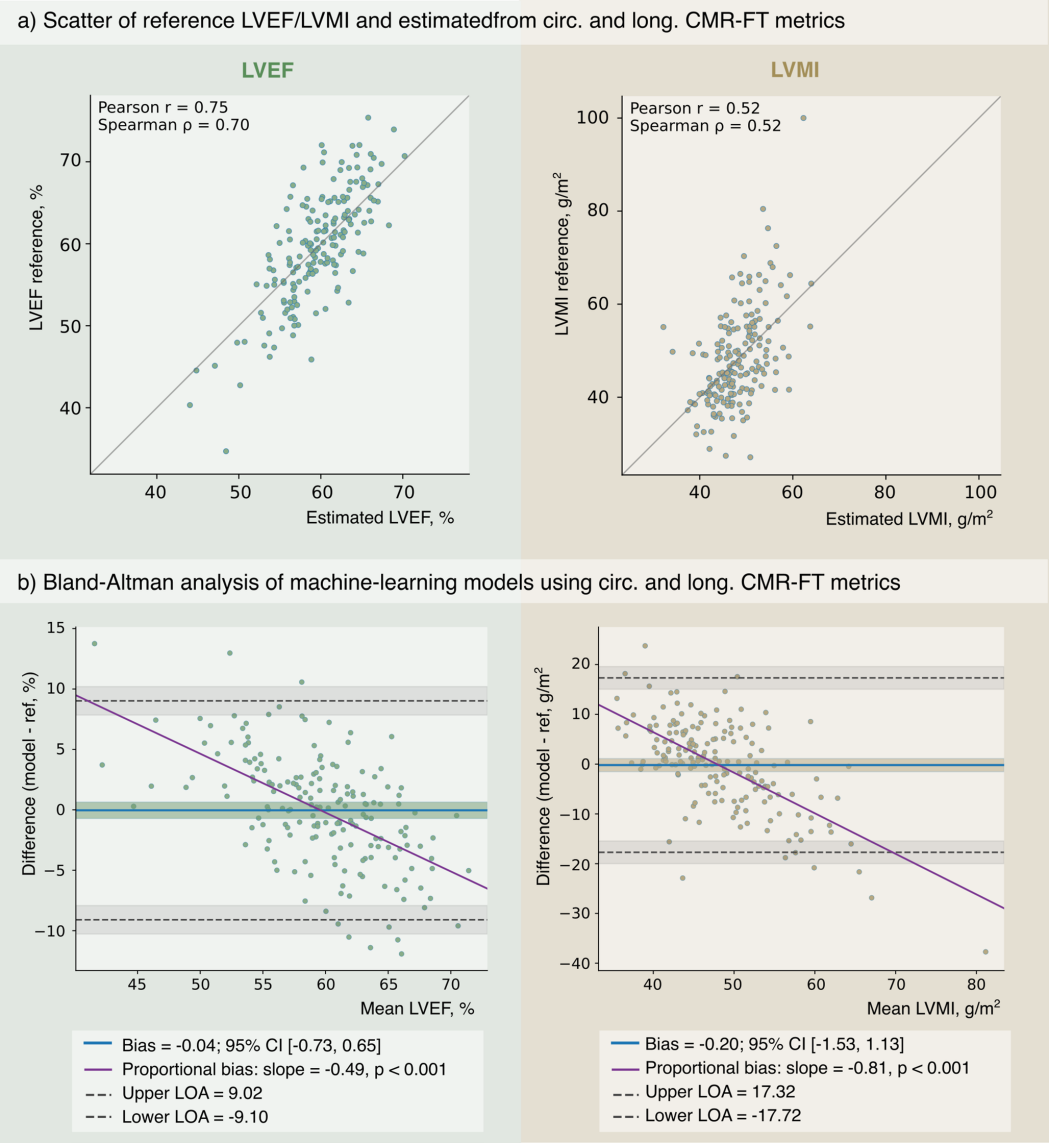
Agreement between model estimates and reference LVEF and LVMI. (**a**) Scatter plots of estimated versus reference LVEF and LVMI with line of identity (**b**) Bland–Altman plots of model estimate and reference LVEF and LVMI

Primary models produced out-of-sample estimates that tracked reference values (Fig. 8), with stronger association for LVEF (r = 0.75, ρ = 0.70) than LVMI (r = 0.52, ρ = 0.52). Mean bias was near zero, but proportional bias was present (both p < 0.001) (Fig. 8b). Across 50 repeats, median absolute error was 2.9 %-points (IQR 1.4 – 5.3) for LVEF and 5.9 g/m^2^ (IQR 2.3 – 9.4) for LVMI. RMSE was 4.6 %-points and 8.9 g/m^2^, respectively. For context, published CMR expert agreement RMSE: 4.2 %-points for LVEF, and 17.5 g for LV mass ≈ 9.2 g/m^2^ for LVMI when indexed to BSA = 1.90 m^2^ (median BSA of current population) [17].

Outcome-permutation yielded estimates clustered around the training-set-mean, and non-significant Spearman rank correlations between predicted and reference LVEF and LVMI (ρ = 0.03, p = 0.708 for LVEF, and ρ = 0.11, p = 0.14 for LVEF) indicating no meaningful signal under shuffled labels and supporting that performance with true labels reflects genuine feature-outcome associations.

### Complementarity of mechanical metrics to clinical variables

**Fig. 9:**
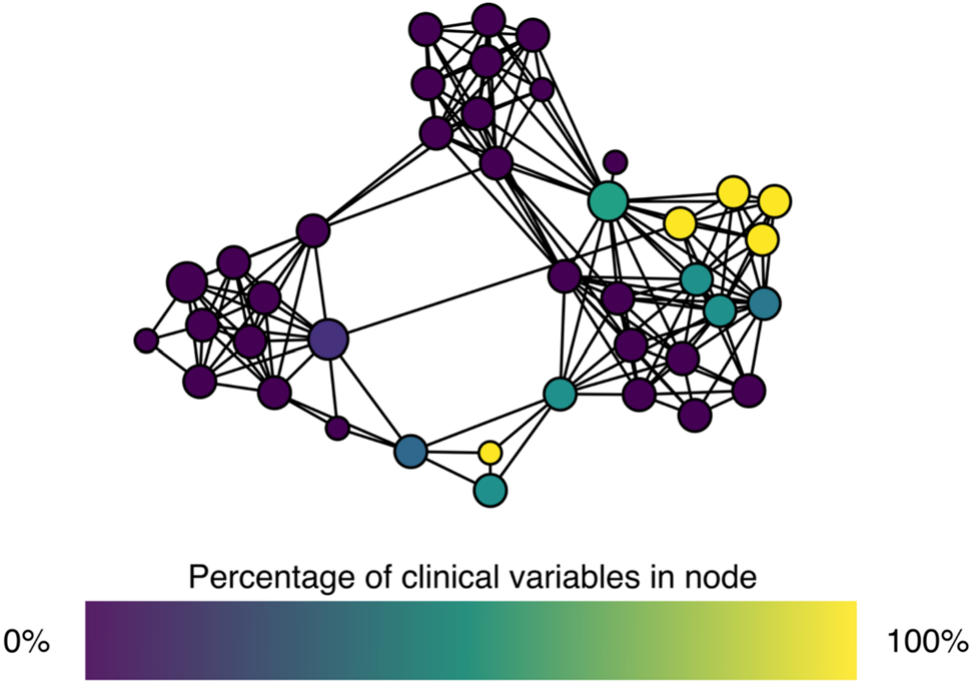
Topological data analysis plot of radial, circumferential, and longitudinal mechanical metrics and clinical variables (age, sex, diabetes, BMI, BSA, hypertension, smoking status) associated with LVEF, LVMI and cardiovascular risk. Nodes represent variable clusters sharing similar traits across participants. Edges connect clusters with overlapping variables. Node colours indicate percentage of clinical variables in each cluster

Figure 9 and supplementary results present informational content of CMR-FT metrics and clinical variables. Clinical variables concentrated in a subnetwork with limited mixing with CMR-FT metrics, indicating largely non-redundant information.

## Discussion

This study has three main contributions. First, it simultaneously evaluates the associations between a broad panel of global CMR-FT mechanical measures and LVEF and LVMI using multivariable modelling and SHAP-based feature ranking, rather than examining selected metrics individually. From these joint models, mechanical phenotypes of LVEF and LVMI are derived, defined as the ranked pattern of mechanical metrics that most strongly shape model estimates, together with the direction of their associations. Second, it provides evidence that multiple CMR-FT metrics beyond GLS carry signal with respect to LVEF and LVMI. This supports their potential utility as candidate features for future diagnostic and prognostic modelling rather than as supplementary outputs or noise. Third, it motivates moving beyond single-metric summaries towards integrated, multivariable characterisation of myocardial mechanics in future studies targeting more clinically actionable diagnostic or prognostic endpoints.

### Mechanical phenotypes of LVEF and LVMI

Higher LVEF was primarily associated with greater circumferential shortening (higher |GCS|) and greater longitudinal systolic strain rate (higher |sSR_long_|) in the joint models, which also ranked highest in univariable analyses, positioning these mechanical properties as dominant discriminants of LV ejection. Although GLS showed a substantial correlation with LVEF, its association when contextualised was comparatively small.

Higher LVMI was most strongly linked to reduced peak diastolic strain rates (|dSR_long_| and |dSR_circ_|) and reduced longitudinal shortening (lower |GLS|), concordant in both joint and univariate analyses. The high impact of diastolic strain rates is consistent with pathophysiological understanding of hypertrophic remodelling being associated with impaired relaxation and passive filling ("stiff myocardium") [22].

Rank differences between univariable correlation and SHAP-based multivariable importance are informative rather than contradictory. The marked drop in GLS’s rank for LVEF suggests that its substantial correlation did not translate into an equally important contextual contribution. This may reflect residual redundancy, as part of the information captured by GLS may overlap with GCS and sSR_long_, but it could also reflect non-additive effects captured by the flexible joint model, giving other mechanical metrics greater contextual importance. Repeated model re-runs showed that GCS consistently ranked highest for LVEF (SD of rank = 0), indicating a reproducibly dominant contextual contribution. In contrast, GLS remained highly ranked for LVMI also when mechanics were considered jointly. This suggests that GLS may retain more signal for LVMI also in a contextualised setting, either because it is less redundant, or because other metrics gain relatively less from contextualised analysis. The present analyses were not designed to disentangle these possibilities.

Consistent with prior studies, worse strain associated with lower LVEF [23–25], and higher LV mass [26]. The present study extends evidence for this and demonstrates associations for a broader panel of myocardial mechanics. Further, the current work showed stronger GCS-LVEF association than GLS-LVEF association, which amplified upon joint modelling. This is concordant with Stokke et al. who derived and empirically validated an analytical model where LVEF depended quadratically on GCS, and linearly on GLS [24]. We observed stronger correlations between both GLS and GCS, and LVEF and LVMI than previously reported by speckle-tracking-echocardiography [23], which may reflect limited interchangeability between modalities [27–29]. The agreement of relative association magnitude and association direction supports external validity and physiological plausibility of the proposed approach for joint CMR-FT modelling, though validation in other cohorts is still needed.

### Beyond single metrics and future utility

While pairwise correlations show stand-alone association between individual CMR-FT metrics and outcomes-of-interest, they do not indicate whether a metric is redundant to other mechanical descriptors, nor capture non-monotonic or joint effects arising from metric-interactions. The flexible joint modelling framework applied here allows modelling of both non-monotonic mechanics-outcome associations and contextual analysis. This provides (i) broader physiological insight about how mechanics relate to outcomes-of-interest (here LVEF and LVMI), and (ii) shows that other CMR-FT metrics, when contextualised, could provide stronger signal than GLS alone. The latter supports using contextual, systems-level characterisation of myocardial mechanics beyond GLS when modelling more actionable outcomes of interest using myocardial mechanics. Although model performance is comparable to expert agreement, estimation of LVEF/LVMI using machine learning models from CMR-derived mechanical metrics is not clinically necessary as CMR is their reference standard. However, the achieved results demonstrate the untapped potential of mechanical metrics that are already provided by clinically available software but seldom leveraged. The key message is that broader mechanical characterisation of the myocardium carries information about established measures of cardiac function and structure. This suggests that such characterisation deserves further investigation in studies with clinically actionable endpoints, rather than implying that specific mechanical metrics should be used as proxies for LVEF or LVMI. Future studies could investigate mechanical signatures of clinically useful outcomes, such as heart failure with preserved ejection fraction (HFpEF), subclinical cardiotoxicity, or risk stratification in hypertrophic cardiomyopathy. The present findings are hypothesis-generating and support further evaluation in studies designed for diagnostic or prognostic assessment.

### Redundancy of radial metrics?

In this study, circumferential and radial strain-derived measures (peak strain, time-to-peak strain, and systolic/diastolic strain rates) were highly correlated, possibly reflecting that both measures are derived from short axis imaging and capture aspects of myocardial deformation and therefore offer limited incremental information. On the other hand, displacement-derived metrics (peak displacement, time-to-peak displacement and peak systolic/diastolic velocities) were less redundant and may offer complementary information, and future studies may explore their combined value. Also, model performance was significantly better for LVMI but not LVEF when radial metrics were included, supporting their addition when model performance, rather than interpretation, is the objective.

### Added value beyond clinical variables

CMR-FT metrics (radial, circumferential, longitudinal) provided complementary information beyond standard clinical variables and cardiovascular risk factors (age, sex, diabetes, BMI, BSA, hypertension, smoking) in the currently studied selected general population (Supplement). This is consistent with the topological data analysis map that demonstrated largely distinct clustering of mechanical metrics from clinical variables, indicating limited information redundancy between those two variable categories. In line with this, models trained on both clinical variables and CMR-FT metrics (radial, circumferential, and longitudinal) had significantly lower errors than models trained on clinical variables only.

### Limitations

The homogenous study sample limits generalisability. Mechanical signatures of LVEF and LVMI could differ in athletes, patients with cardiac diseases, and if obtained through different pipelines. The present analyses do not determine whether the relative importance of mechanical metrics differs between women and men. Further, mechanical metrics can vary with field strength, vendor, post-processing software as well as across imaging modalities [27–30]. Nonetheless, the presented approach for mechanical insight is scalable/adaptable to other populations and pipelines.

Given the cross-sectional design causality cannot be established. Results are regarded associational/hypothesis generating.

## Conclusion

This study identified integrated mechanical CMR-FT profiles of LVEF and LVMI among older adults from the general population. SHAP analyses of multivariable models reordered feature importance relative to univariate correlations, showing that metrics highly correlated with LVEF or LVMI may not provide the most independent information once considered in the context of others. This suggests that cardiac function characterisation could be reframed around integrated, interpretable, system-level mechanics. Future work should test whether integrated mechanical profiles add prognostic and diagnostic value beyond single-metric markers.

## Supplementary Information

Below is the link to the electronic supplementary material.

Supplementary file1

## Data Availability

The data that support the findings of this study are derived from the ACE 1950 CMR substudy and are not publicly available due to participant privacy protections and restrictions under the study’s data governance approvals. De-identified data may be made available upon reasonable request, subject to approval by the ACE 1950 study steering committee.
