# Supplementary material for: Mechanical signatures of left ventricular ejection fraction and mass index in a community cohort (ACE 1950)
Source: Int J Cardiovasc Imaging. 2026 Apr 27;42(8):1505–22. doi: 10.1007/s10554-026-03697-7 (PMC13433544; doi:10.1007/s10554-026-03697-7)
Supplement: Supplementary file 1 [file 10554_2026_3697_MOESM1_ESM.pdf]

Supplementary material

Supplementary material of article "Mechanical signatures of left ventricular ejection fraction and mass index in a community cohort (ACE 1950)"

Supplementary method

Assessing added value of CMR-FT metrics to clinical variables

Additional sensitivity models were trained using (i) seven clinical variables known to be linked to LVEF, LVMI or cardiovascular risk, (ii) all 25 (radial, circumferential and longitudinal) CMR-FT metrics, (iii) a combination of the clinical variables and the 25 (radial, circumferential and longitudinal) CMR-FT metrics (in total 32 inputs). The clinical input features were sex, age, hypertension (defined as daily use of antihypertensive medication, or systolic blood pressure ≥ 140 mmHg, or diastolic blood pressure ≥ 90 mmHg), body mass index (BMI), body surface area (BSA), diabetes mellitus (self-reported, or use of antidiabetic medication, or HbA1c ≥ 6.5 %, or fasting blood glucose ≥ 7.0 mmol/L [126 mg/dL]), and smoking status (as categorical variable obtained from questionnaires: daily smoking vs not daily smoking).

Statistical comparisons

Differences in the absolute estimation errors between models trained on mechanical metrics only and the additional models trained on clinical variables only and a combination of clinical and mechanical metrics were assessed using the Friedman test. Pairwise comparisons were then conducted with the Wilcoxon signed-rank test (null hypothesis: no difference in absolute error). To control the Type I error rate upon multiple comparisons (across multiple feature models) the Holm's correction was applied, as recommended by Demšar (1).

Supplementary results

Complementarity of mechanical metrics to clinical variables

There was no significant difference in absolute error between the mechanics-only and the combined model for LVEF ($p = 0.054$) or LVMI ($p = 0.217$) estimation. Further, both the mechanics-only and the combined models outperformed the clinical-only model for both LVEF and LVMI estimation. For LVEF, the clinical-only model had a median absolute error of 4.2 percentage points, IQR [1.9, 7.9], higher than mechanics-only 2.7 percentage points, IQR [1.5, 5.2] and combined 2.6 percentage points, IQR [1.5, 4.7] (both $p < 0.001$ vs clinical-only). For LVMI, the clinical-only model had a median absolute error of 5.5 g/m^2, IQR [2.7, 8.6], whereas mechanics-only and combined models achieved 4.1 g/m^2, IQR [1.8, 7.3] and 4.1 g/m^2 IQR [1.8, 7.4], respectively (paired Wilcoxon $p = 0.007$ and $p < 0.001$ vs clinical-only).

RMSE showed the same pattern with higher errors made by the clinical-only models compared to the mechanics-only and combined models both for LVEF (RMSE 4.6 percentage points in the clinical model and 3.0 percentage points in the mechanics-only, and 3.1 percentage points in the combined), and for LVMI (RMSE 5.7 g/m^2 in the clinical model and 4.9 g/m^2 in the mechanics-only, and 4.7 g/m^2in the combined).

How sex associated with LVEF and LVMI

Interpreting the clinical and combined machine-learning models with SHAP revealed that female sex was associated with higher estimated LVEF and lower estimated LVMI, consistent with established sex differences in CMR reference values (2). Among the 32 radial, circumferential, and longitudinal mechanical metrics and clinical variables, sex ranked as 5th most impactful variable for LVMI and 15th for LVEF.

References of the supplement

1. Demšar J. Statistical Comparisons of Classifiers over Multiple Data Sets. Journal of Machine Learning Research. 2006.
2. Kawel-Boehm N, Hetzel SJ, Ambale-Venkatesh B, Captur G, Francois CJ, Jerosch-Herold M, et al. Reference ranges ("normal values") for cardiovascular magnetic resonance (CMR) in adults and children: 2020 update. J Cardiovasc Magn Reson. 2020;22(1):87.
